# Prevalence of intestinal parasites among HIV patients in Baringo, Kenya

**Published:** 2012-10-21

**Authors:** Cornelius Kibet Kipyegen, Robert Shavulimo Shivairo, Rose Ogwang Odhiambo

**Affiliations:** 1 Department of Biological science, Egerton University; 2 Department of Animal Health, Egerton University, Kenya

**Keywords:** Prevalence, intestinal parasites, HIV, Kenya

## Abstract

**Introduction:**

HIV patients have reduced immune response which makes them more susceptible to different infections. This cross-sectional study was carried out to document the prevalence of intestinal parasites among HIV patients in Baringo County, Kenya.

**Methods:**

Structured questionnaires were used to collect clinical information after obtaining consent from the participants. Stool samples were collected from 285 respondents for intestinal parasitic examination using direct and formol-ether concentration to detect ova and cysts. Chi-square (X^2^) statistical analysis was used to test level of significance at P = 0.05 using SPSS.

**Results:**

A prevalence of 50.9% of intestinal parasites was recorded. Majority of the parasitic infections were waterborne protozoa with few helminthes. There was an association (P < 0.05) between intestinal parasitic infection and place of residence, agro-ecological location, family size, water source, treatment and reliability and diarrheal status. There was no association (P > 0.05) between age groups and gender with parasitic infection. Parasites identified were *Entamoeba histolytica/dispar* (58.3%), *Giardia lamblia* (16.6%), *Ascaris lumbricoides* (8.6%), *Entamoeba coli* (5.9%), *Taenia saginata* (5.3%), *Trichuris trichuria* (1.9%), *Enterobius vermicularis* (1.9%) and hookworm (1.3%).

**Conclusion:**

There was high prevalence of intestinal parasites, therefore, health education to HIV patients and community health workers on the importance of good environmental sanitation and personal hygiene could curb water, food and individual contamination promoting good management and care of HIV patients, hence improving their health status.

## Introduction

HIV/AIDS has become a major public health concern in the African continent accounting for 67% of infection worldwide and in Kenya, there is a prevalence of 7.8 percent among 15-49 year olds individuals [1]. Gastrointestinal problems resulting from opportunistic parasitic infections in HIV and AIDS infected subjects often present as diarrhea and significant disease has been recorded in 50-96% of cases worldwide with 90% prevalence rate reported in Africa [2]. In developed countries diarrhea occurs in 30-60% of AIDS patients and 90% in the developing countries [3]. Acute and chronic diarrhea has been associated with different species of gastrointestinal parasites, which are responsible for considerable morbidity and mortality in HIV/AIDS patients [4].

Intestinal parasitic infections play an important role in the progression of HIV infection, by further disturbing the immune system while it is already engaged in the fight against HIV [5]. The gastrointestinal pathology associated with HIV infection comprises significant enteropathy with increased levels of inflammation and decreased levels of mucosal repair and regeneration [6]. HIV infection leads to loss of CD4^+^T cells, which leaves affected individuals mortally susceptible to opportunistic infections. Many of the opportunistic infections that ultimately plague such individuals involve infectious agents that are normally checked by the mucosal barriers which include *Cryptosporidium spp, Giardia lamblia, Entamoeba histolytica, Ascaris lumbricoides*, hookworm infection, *Schistosoma spp* and *Strongyloides stercoralis* are important cosmopolitant intestinal parasites that are common among children and immunocompromised individual [7]. The pathogenic intestinal parasites such as *Cryptosporidium, Cyclospora, E. histolytica* and *Giardia*, can last for months in patients with AIDS, causing malabsorption of nutrients, gradual debilitation through dehydration, and metabolic abnormalities and are responsible for severe diarrheal episodes [8, 9].

The sources of parasitic infections in humans include contaminated soil, food and water sources with human faeces and poor sewage disposal such as use of night soil as fertilizer [8, 10]. Fecal oral route is significant in the transmission of parasitic infections via poor personal hygiene and environmental contamination. Nosocomial outbreaks of cryptosporidiosis have also been described, where an individual gets infected in hospital [8].

In developing countries, gastrointestinal parasite infections are mostly due to poverty characterized by poor hygienic habits, absence of portable and clean water, absence of good faecal disposal system and poor nutrition. Pathogens responsible for causing diarrhea differ according to geographical location, therefore laboratory diagnostic evaluations are required to determine their prevalence in each population so as to provide guidelines for therapy and necessary data for planning and evaluation of HIV-positive/AIDS patients care and management. Furthermore, areas with different agro-ecological zones could favour the development of different intestinal parasites. The study area is divided into three agro-ecological zones; highland, midland and lowland, each with different climatic conditions. Therefore, documentation of distribution of intestinal parasites in these three zones is important because the information generated will be useful for implementation of the control and prevention of intestinal parasitic diseases in the area. The aim of this study was to document the prevalence of intestinal parasites among HIV-positive patients in Baringo District, Kenya.

## Methods

### Study area

Baringo County covers approximately an area of 8,655 Km^2^ and is divided into three agro-ecological zones namely the highlands, midlands and lowlands. The highlands are characterized by the Tugen hills, which rise to an average of 2,000 meters above the sea level. The annual average rainfall is 1,200mm, and annual average temperature is about 25°C. The midlands are inhabited by the agro-pastoralists; this zone is endowed with the three rivers in the district namely the Perkerra, Molo and Kerio Rivers. The lowlands have an average altitude of about 700 meters above the sea level and most of it is rangeland. Temperatures in this zone are above 32°C, and the average rainfall of 600mm. Settlement patterns in the district are determined by climatic condition and economic opportunity of the area.

### Research design

This was a cross-sectional survey that involved interviewing of the HIV/AIDS patients using structured a questionnaire and also laboratory analysis of stool specimen for protozoa and helminth from the respondents. The structured questionnaire enabled the acquisition of information of the participants. It contained three sections which include: Demographic and socio-economic information (age, gender, residence, location, family size and occupation of parents), Environmental factors (housing conditions and water supply) and health (history of intestinal parasitic infection and diarrhea). The laboratory analysis was carried out by stool analysis using microscopy, to diagnose for intestinal parasitic infections.

### Study population

HIV/AIDS patients who were registered clients of Academic Model Providing Access to Healthcare (AMPATH) clinic at Baringo District Hospital were the study population. The inclusion criteria were: patients who were HIV-positive, with or without signs of diarrhea, willing to participate in the study and had ability to comprehend the importance of the study. A total of 285 HIV positive patients were included in the study consisting of 94 male and 191 female. The distribution of the participant across the agro-ecological zones was done using simple random sampling. The exclusion criteria were: those on specific antihelminthics or who had any treatment for intestinal parasitism in the last two weeks preceding specimen collection and those who had antacid, in the last two weeks preceding specimen collection were all excluded from the study because the drugs could have killed or inhibit reproduction of the parasites. The use of medications such as antacids can distort protozoan morphology contributing to the difficulty in identifying the organism. The study group included the HIV-positive patients with diarrhea and the control group included the HIV positive patients without any abdominal complain.

### Ethical Considerations and Treatment

Verbal and written informed consent was sought from the participants after informing them about the importance of the study and their rights to decline participation in the study before inclusion in the study. For those of age below 18 years, consent was obtained from their parents/guardians before recruiting into the study. The ethical approval of the study was sought and obtained from the Moi Teaching and Referral Hospital and Moi University Institutional Research and Ethical Committee (IREC). All HIV positive patients with positive stool analysis were appropriately treated free of charge.

### Specimen Collection and Processing

Stool specimens were collected using a clean wide mouth specimen container from patients attending hospital for clinical treatment. Freshly voided stool specimens were collected, processed and examined microscopically as saline wet mount to detect larva, eggs, trophozoites and cysts of the parasites [11]. Also, Formol-ether concentration was performed and modified cold Zeihl-Neelsen (ZN) was used to detect coccidian species.

### Data analysis

The data obtained from questionnaires was analyzed using the statistical software SPSS version 11.5. The information obtained from the questionnaire was presented as percentages and means. The results obtained from the stool specimens were presented as frequency and percentage. Chi-square test was used to determine the association between different variables in the structured questionnaire and intestinal parasitic infection. The results were statistically significant if the p-value was less than 0.05 and vice-versa.

## Results

### The prevalence rates of Intestinal parasites in HIV patients in Baringo

The prevalence of intestinal parasites was 50.9%. Majority of the intestinal parasites were intestinal protozoan which recorded a prevalence of 80.8%, with the *E. histolytica/dispar/moshkovskii* having frequency of 88 (58.3%), while *G. lamblia* and *E. coli* recorded 25 (16.6%) and 9 (5.9%) respectively. The helminth with highest prevalence was *A. lumbricoides* which had frequency of 13 (8.6%), while Hookworm was the least with 1.3% (Table 1).

**Table 1:** Distribution of intestinal parasites among HIV positive patients

Parasite	Frequency of parasites	Proportion (%)
*E. istolytica/dispar*	88	58.3
*G. lamblia*	25	16.6
*A. lumbricoides*	13	8.6
*E. coli*	9	5.9
*Teania* spp	8	5.3
*E. vermicularis*	3	1.9
*T. trichuria*	3	1.9
Hookworm	2	1.3
Total	151	100.0

Majority of the parasitic infection were intestinal protozoans. *E. histolytica/dispar* having highest frequency. Few helmithic infections were recorded with *A. lumbricoides* and Taenia species being predominant.

### The prevalence of infection with respondent's characteristics

There was no significant association between age and parasitic infection (X^2^=3.123, df = 5, P > 0.05). Those among the age group 20-39 years recorded the highest prevalence of intestinal parasitic infection of 26.7%, while the age group of above 60 years recorded the least prevalence 1.1% (Table 2).

**Table 2:** Demographic information and prevalence of intestinal parasites in HIV positive patients in Baringo

Characteristics	Number examined	Infection status		P-value
	Frequency (%)	Infected	Uninfected	
**Age (Years)**				
0-5	17 (6.0)	7 (2.5)	10 (3.5)	0.681
6-12	27 (9.5)	14 (4.9)	13 (4.6)	
13-19	9 (3.2)	3 (1.1)	6 (2.1)	
20-39	151 (53.0)	76 (26.7)	75 (26.3)	
40-59	77 (27.0)	42 (14.7)	35 (12.3)	
>60	4 (1.4)	3 (1.1)	1 (0.4)	
**Gender**				
Male	94 (33.0)	46 (16.1)	48 (16.8)	0.646
Female	191 (67.0)	99 (34.7)	92 (32.3)	
**Family size**				
1-3	43 (15.1)	15 (5.3)	28 (9.8)	0.05
4-7	165 (57.9)	86 (30.2)	79 (27.7)	
>8	77 (27.0)	44 (15.4)	33 (11.6)	
**Place of residence**				
Urban	80 (28.1)	29 (10.2)	51 (19.9)	0.002
Rural	205 (71.9)	116 (40.7)	89 (31.2)	
**Diarrheal status**				
Positive	175 (61.4)	125 (43.9)	50 (17.5)	0.0001
Negative	110 (38.6)	20 (7.0)	90 (31.6)	

Individuals between the age group 20-59 which forms the active group were the most infected with HIV and also recorded high parasitic infection rate. Female were more infected with HIV than men and this could be as a result of biological or cultural factors. Those who had diarrhea had high intestinal parasitic infection than those who had no diarrhea.

In relation to gender, there was no significant difference (X^2^=0.211, df = 1, p > 0.05). The male had an infection of 16.1%, while 34.7% of the infected being female. There was a significant association between intestinal infection and the place of residence (X^2^=9.521, df = 1, P < 0.05). Those residing at rural areas were most infected with the prevalence of 40.7%, while the urban residence had a prevalence of 10.2%. Diarrhea was associated with intestinal parasitic infection, X^2^=76.624, df = 1, P < 0.05. Out of 61.4% of the HIV positive patients who presented with diarrhea 43.9% were infected with intestinal parasites with 17.5% having no parasitic infection, while those who had no diarrhea 7% were positive for intestinal parasitism and 31.6% were not infected.

There was a significant difference between agro-ecological location and intestinal parasitic infection (X^2^=22.14, df = 2, P < 0.05) (Table 3). Those who live in the lowlands recorded a high prevalence of 20.0%, and those living in the midlands had the least 14.0%. A cross tabulation of the infection of intestinal parasites, agro-ecological location and place of residence of the respondents indicates an association of infection among those in lowlands (X^2^=9.375, df = 1, P < 0.05). There was no association in the highlands (X^2^=0.196, df = 1, P > 0.05) and midlands (X^2^=0.436, df = 1, P > 0.05).

**Table 3:** Place of residence, location of respondent and infection status

		Infection status			
Agro-ecological zone	Place of residence	Infected (%)	Uninfected (%)	Total (%)	P-Value
Highland	Urban	19 (6.7)	37 (13.0)	56 (19.7)	0.658
	Rural	29 (10.2)	48 (16.8)	77 (27.0)	
Midland	Urban	4 (1.4)	1 (0.4)	5 (1.8)	0.509
	Rural	36 (12.6)	19 (6.7)	55 (19.3)	
Lowland	Urban	6 (2.1)	13 (4.6)	19 (6.7)	0.05
	Rural	51 (17.9)	22 (7.6)	73 (25.5)	
	Total	145 (50.9)	140 (49.1)	285 (100)	

Participants from the lowlands had high intestinal parasitic infection due to unreliable water which compromises the level of sanitation and hygiene. Infection among those in highland is because of water source (river) which is not safe and could be contaminated at the source or during transportation.

### Sources of water in relation with infection status

There was a strong association between infection status with respondents sources of water (X^2^=19.236, p < 0.05) (Table 4). Respondents who obtain water from the river were more likely to be infected by the intestinal parasites. Respondents obtaining water from bore hole were less likely to be infected with the parasites. There was a significance difference of parasitic infection with the reliability of water (X^2^=4.223, df = 1, p < 0.05). Infection was high among those who mentioned that their water source was not reliable. Inadequate water availability could lead to poor sanitation posing a risk to parasitic infections. High infection rates were associated with lack of water treatment practices among the respondents (X^2^=40.333, df = 1, p < 0.05), where an individual can get exposed to contaminated water with cyst/ova of the intestinal parasites.

**Table 4:** Source of water, reliability and treatment in relation with infection status

	Infection status			
Source of water	Infected	Uninfected	Total	P-Value
Piped water	19 (6.7)	44 (15.4)	63	0.0001
Bore hole	5 (1.8)	6 (2.1)	11	
Bottled water	8 (2.8)	4 (1.4)	12	
River	102 (35.8)	84 (29.5)	186	
Tank	11 (3.8)	2 (0.7)	13	
Total	145 (50.9)	140 (49.1)	285	
**Water reliability**				
Reliable	50 (17.5)	65 (22.8)	115	0.04
Not reliable	95 (33.3)	75 (26.3)	170	
Total	145 (50.8)	140 (49.1)	285	
**Treatment of water**				
Treat	37 (13.0)	88 (30.9)	125	0.0001
Do not treat	108 (37.9)	52 (18.2)	160	
Total	145 (50.9)	140 (49.1)	285	

Source of water play a major role in parasitic infections, those who obtained water from river had high parasitic infection due contamination at water point by both animals and human. Treatment of water is before consumption is crucial which was not the case, as majority of infections was among those who don't treat water.

## Discussion

HIV and AIDS patients have reduced immune response, thus are at high risk of acquiring gastrointestinal parasitic infections which are responsible for considerable morbidity and mortality in HIV and AIDS patients [4]. The distribution of intestinal parasites varies according to the geographical location due to different climatic conditions. In Kenya, majority of the population are rural dwellers stricken with poverty characterized by poor hygienic habits, absence of potable and clean water, absence of good faecal disposal system, low level of education and poor nutrition, hence increasing the rate of infection of intestinal parasites. This study was carried out in Baringo County where most of the participants are from the rural areas with a few living in the peri-urban.

In this study, there was high prevalence of intestinal parasites in HIV patients and this is as a result of reduced immune response making them susceptible to opportunistic infections. The prevalence of intestinal parasites among HIV patients in this study is in consistency with similar studies carried out in Ethiopia (52.6%) [12], (59.8%) [13], Turkey (47%) [14], Thailand (50%) [15] and India (50 to 80%) [16]. Majority of the intestinal parasites were the protozoan with few helminths present. The most prevalent protozoan parasite is *E. histolytica/dispar*, while *A. lumbricoides* was the prevalent helminth (Table 1). Majority of these infections were found in rural areas where there is poor personal hygiene and poor environmental sanitation leading to contamination of soil and water sources. The inhabitants of rural areas obtain water from the river and according to this study it was found most of the respondents don't treat water before drinking exposing them to high risk of using contaminated water directly [17]. Few infection cases were recorded amongst peri-urban dwellers, because they had access to piped water which is already treated thus get less infected if they handle the water safely, however, it has been noted that treated water could still harbor parasites, especially cyst of *G. lamblia*which is resistant to standard water treatment [18].

According to the demographic characteristics of the HIV patients (Table 2), it was found that intestinal parasitic infection was highest in respondents aged between 20-39 years and 40 - 49 years and this is the age category most infected with HIV in rural areas in Kenya [1, 19]. There was no association between respondent's age and parasitic infection (P > 0.05), this could be as a result that all participants had HIV infection hence reduced immunity which then makes them vulnerable to intestinal parasitic infection [20]. In relation to gender, there was no significant difference (P > 0.05) of parasitic infection. The high prevalence in female could be because of the household activities that they engage in like child care, constant contact with water and contaminated soil most of the time more than men as they perform domestic chores and agricultural activities, hence more vulnerable to parasitic infection [21].

Intestinal parasitic infection in relation to the agro-ecological location of the participants in Baringo District shows that those living in the lowlands had a high prevalence with those in midlands being least infected (Table 3). The environmental conditions which include temperature, wind and humidity in the midlands and lowlands favor the viability and maturation eggs of helminths and also the cyst of the intestinal protozoan are relatively resistant against adverse environment which leads to high prevalence of infection [22]. Majority of the residents in these two zones are illiterate because they engage mostly in pastoralist with most of population not attending school, thus have little information on the importance of sanitation. This also poses the risk of zoonotic transmission of intestinal parasites because they come into contact with their animals or even environmental contamination especially the water points. There is also the problem of accessing clean water for drinking and household use posing a risk of infection with intestinal parasites.

Family size was significantly associated with intestinal parasite infection (P < 0.05), large family size were more likely to be infected than those with small families (Table 2). This could be as a result of congestion in the house which can compromise sanitation and nutrition. This also determines the highest level of education an individual can acquire because in large family the expenditure on education will be high making other not to afford. Low level of education especially in parents could lead to intestinal parasitic infection [23], because they lack information on the importance of practicing good hygiene and sanitation.

There was a strong association between infection status with respondents sources of water (P < 0.05) (Table 4). Respondents who obtain water from the river were more likely to be infected by the intestinal parasites with. Those obtaining water from bore hole were less likely to be infected with the parasites. The source where drinking water is taken has important value to health and to the quality of life. Sources like rivers, lakes, and even groundwater from which drinking water is taken may contain a wide variety of contaminants, both chemical and microbial, that can cause illness and disease [17, 24]. These water sources can be contaminated with parasitic elements that cause disease due to various unhygienic practices like defecating around water sources. These elements can then get their way back to water sources when washed down by with rain water leading to water contamination. Basic hygiene is impossible to be achieved if there is insufficient quantity of water. Reliability of the water source was significantly associated with infection status (P < 0.05). According to this study, it was found that there was an association between sources of water and water treatment (P < 0.05). Water source in relation to treatment shows that respondents who obtain water from river do not treat which explains why individual who obtain water from river had high intestinal parasitic infections. Safe treatment and storage of water at the point of use has been shown to reduce the risk of diarrheal disease by 30 to 40% [25] especially in HIV patients.

HIV patients who were examined positive for intestinal parasitic infection were treated accordingly. Patients found to be harboring intestinal protozoan were administered with metronidazole, while those who had helmithic infection were given mebendazole. Patients who were severely dehydrated due diarrhea were given intra venous rehydration. All HIV patients regardless of their immunological status, ARV drug treatment or age were administered with Cotrimoxazole. This drug is effective against some bacterial infections, malaria and diarrhea causing protozoans.

## Conclusion

There was high prevalence of intestinal parasites among HIV positive patients, with most of the parasites found being waterborne protozoan. Provision of safe drinking water and health education on efficient environmental sanitation and improving personal hygiene could enable curb water, food and individual contamination, thus promoting good health. This study is in line with the Kenya National AIDS Strategic Plan (KNASP III) which aims to achieve universal access to services targeting for quality integrated services to reduce HIV related illnesses and deaths and prevent new infections. This will lead to the realization of Kenya vision 2030 and also achievement of the Millennium Development Goals (MDG).
